# Supplementary material for: Are carbon emissions trading and green financial instruments synergistic? -Comprehensive quantitative research based on content analysis
Source: PLoS One. 2024 Mar 7;19(3):e0298601. doi: 10.1371/journal.pone.0298601 (PMC10919601; doi:10.1371/journal.pone.0298601)
Supplement: S1 Data — (ZIP) [file pone.0298601.s001.zip › Data/地级市Z_F工作报告.zip!/╦─┤¿/└╓╔╜╩╨/└┤╫╘╣1⁄2╓┌║┼í╛┬φ┐╦╩2╛▌═°í┐_2018.docx]

各位代表：

　　现在，我代表乐山市人民政府，向大会报告工作，请予审查，并请市政协各位委员提出意见。

　　2017年工作回顾

　　2017年是新一届政府的开局之年。一年来，我们深入学习贯彻党的十八大、十九大精神，以习近平新时代中国特色社会主义思想为指导，在市委的坚强领导下，在市人大、市政协的监督支持下，紧紧围绕建设四川旅游首选地、绿色转型示范市、山水园林宜居城、总部经济聚集区发展定位，紧扣“基础设施建设年”工作主题，统筹稳增长、促改革、调结构、惠民生、防风险各项工作，经济社会发展取得新的成绩。预计(下同)全市实现地区生产总值1525亿元、增长8.2%左右;全社会固定资产投资1254亿元、增长13%;规模以上工业增加值增长9.5%;社会消费品零售总额705.5亿元、增长13%;地方一般公共预算收入98.9亿元、增长8.5%;城镇居民人均可支配收入30955元、增长8.3%，农村居民人均可支配收入13947元、增长9.4%;主要经济指标增速均高于年初预期、高于全省平均水平。

　　一年来，我们重点抓了以下八个方面工作：

　　一是挂图作战推进项目。我们按照全省“项目年”部署，挂图作战抓项目促投资，296个省市重大项目实现投资516亿元，57个挂图作战项目实现投资265亿元。交通建设取得突破，完成交通投资205亿元，绕城高速、井沙联网畅通工程、乐沙城际生态大道、成昆铁路成峨段等项目竣工，乐山机场试验段、乐西高速马边至昭觉段、成乐高速扩容、岷江航电龙溪口枢纽等项目开工。产业化项目有序推进，福华8万吨甘氨酸、川南减震技改搬迁、农夫山泉茶饮料技改等项目竣工投产，沙坪二级水电站首批机组并网发电。城建项目加快实施，完成城建投资260亿元，“一城两新区”基础设施建设有序推进，青衣江大道北段、嘉瑞大道、瑞祥路、大渡河大桥加宽改造、市长途客运车站等项目竣工投用。

　　二是调整优化产业结构。我们积极适应经济发展新常态，主动调结构、转方式、促升级，启动实施服务业倍增计划，净增规模以上服务业企业20家、总数达184家，服务业增加值占地区生产总值比重提高2个百分点以上。旅游主导地位得到强化，全市实现旅游综合收入767.7亿元、增长22.7%，接待国内外游客5077万人次、增长15.9%。成功举办第四届旅博会、第二届茶博会、佛光花海音乐节等会展节庆和第七届世界传统武术锦标赛、2017乐山国际马拉松等高端赛事，“夜游三江”精彩亮相，开启全天候旅游新模式。工业转型步伐加快，新兴产业主营业务收入占规模以上工业比重突破40%。“一总部三基地”实现“一年拉框架”目标，工业集中度提高到75%。规模以上工业企业新增55家，德胜集团、福华农科跻身2017中国民营企业500强。建筑业健康发展，产值突破200亿元。品牌战略深入实施，新增中国驰名商标1件、国家地理标志商标2件、四川名牌8个，5个区县创建为省级知名品牌示范区。农业发展提质增效，绘制发布全省首个“土壤地图”，成功申报峨眉山国家现代农业产业园，发展高标准茶园5万亩，茶业综合产值占到全省四分之一。新增各类农业经营主体339个、“三品一标”农产品86个，产业化带动面达到70%。新型城镇化实现突破，新增城镇建成区面积4.2平方公里，常住人口城镇化率达50%以上，我市进入城市型社会。

　　三是精准发力脱贫攻坚。我们把脱贫攻坚作为头等大事，相继打响春季攻势、夏季战役、秋季攻坚和冬季冲刺，沐川县摘帽通过初审，97个省定和77个市列贫困村退出、38275名贫困人口脱贫通过验收，全市贫困发生率降至1.98%，脱贫攻坚实现再战再胜。创新帮扶举措，出台系列政策措施支持沐川高质量脱贫摘帽，“栋梁工程”等19项社会援助行动有效开展，累计整合投入各类扶贫资金达120亿元。持续改善基础条件，完成易地扶贫搬迁17814人，竣工彝家新寨住房1901户，农村危旧房改造3572户，解决1.9万名贫困人口安全饮水问题，退出贫困村卫生室达标率、义务教育达标率、基本医疗保险参保率均达100%。深入开展“百企帮百村”，570家企业(合作社)与469个贫困村结对，带动发展产业基地33.8万亩，惠及贫困群众1.5万户。组建教育救助、卫生救助、村级产业扶持和小额信贷分险四项扶贫基金，扶贫贷款余额达到66.5亿元，其中扶贫小额信贷余额6.3亿元。实施“集中供养+居家救助”，惠及困难群众3683人。试点实施劳动收入奖励计划、青少年教育促进计划，新增市级以上“四好村”348个，组织贫困劳动力转移就业近1.7万人，励志扶贫奋进行动获中央和省级主要媒体点赞。落实浙川东西扶贫协作帮扶项目38个，帮助美姑县建设扶贫新村4个，乐山一中首期“美姑班”开班。

　　四是动真碰硬保护生态。我们深入贯彻落实中央和省委关于生态文明建设和环境保护系列战略部署，强化政治担当，着力攻坚克难。加强生态保护，初步划定了生态保护红线。以中央和省环保督察为契机，坚持立说立行、边督边改、常态监督，土法造纸作坊污染等一批突出问题得到彻底解决，自然保护区、风景名胜区等重点区域一批环境问题得到有效整治，“环保曝光台”、饮用水水源地迁建和保护等工作获环保部充分肯定。全面打响大气、水、土壤和固体废弃物污染防治“四大战役”，较好完成了“大气十条”第一阶段任务和“水十条”“土十条”年度任务，主要流域水质稳定改善。深入开展绿秀嘉州行动，新增营造林50万亩，全市森林覆盖率57.2%。实施《乐山市中心城区绿心保护条例》，完成绿心生态搬迁1486户。全面推行河长制，市境内河湖堰渠实现河段长全覆盖。“一湖五湿地”建设有序推进，桫椤湖湿地公园通过国家验收，大渡河湿地公园跻身国家试点。

　　五是纵深推进改革开放。我们坚持从改革抓起、以改革破题，累计出台各类改革方案130项，42项国省改革试点取得积极进展。供给侧结构性改革扎实推进，淘汰落后工业企业13家，关闭小煤矿10家、去产能189万吨，拆除“地条钢”生产装备12套，淘汰落后钢铁产能52万吨。旅游综合改革不断深化，综合执法改革启动实施，出台加快旅游业发展十条政策，兑现入境游奖励189.7万元。农业农村改革进展顺利，全市土地确权颁证率达91%，累计流转土地面积65.1万亩，适度规模经营率提高到22%。“多证合一、一照一码”改革落地实行，新登记企业5429家、增长46%。投融资改革取得实效，新组建7个国企集团，完成融资112亿元;105个项目进入财政部PPP项目库，入库量居全省前列;振静股份主板上市，主板上市企业达6家。盐业监管体制改革、中心城区出租汽车经营权改革等平稳实施。创新创业呈现新活力，新获批2家省级中小企业创业示范基地，新培育国家高新技术企业9家、达78家，乐山国家农业科技园区总园落户高新区，成立全国首个彝语智能语音研究院。开放合作成效明显，先后赴巴黎、香港、西安、成都等地开展宣传营销和经贸对接活动，引进世界500强、中国500强及行业前三强企业20家，正式签约项目800亿元，引进到位市外内资630亿元，实际利用外资3000万美元。

六是全面发展社会事业。我们坚持普惠性、均等化、可持续方向，不断完善公共服务体系。教育事业优先发展，教育质量稳步提升，开工建设教育基建项目356个，学前教育普惠率提高到82%，3个县通过义务教育均衡发展省级督导评估，乐山职业技术学院完成整体搬迁，建成农村教师周转房620套、学生宿舍1.9万平方米。卫生健康事业稳步发展，省级卫生城市通过复审，建成医养结合机构13家、养老机构13家，公立医疗机构全部执行药品采购“两票制”，8家三级医院接入国家异地就医联网结算平台。人口自然增长率为2.65‰。文化惠民工程深入实施，老霄顶古建筑群修缮完工，沫若书院揭牌成立，首届“嘉乐汇”戏剧周精彩上演。建成村综合文化服务中心169个，创建省文化扶贫示范村7个。获第四届四川省文明城市称号。成功举办市第七届运动会和市第二届残运会。广播影视、民族宗教、妇女儿童、地方志等工作协调发展。

七是以人为本改善民生。我们始终坚守底线、突出重点、完善制度、引导预期，全面完成十项民生工程和24件民生实事。城镇新增就业5.1万人，城镇登记失业率控制在4.14%。全面完成全民参保登记工作，城乡居民低保标准、退休人员基本养老金、村级干部待遇等调整提高。危旧房棚户区改造开工1.9万户、基本建成2.8万套，货币化安置率100%。新改建公厕30座、旅游厕所150座、停车场10个。新改建农村公路1600公里，改造升级农村电网3492公里。定制推出“菜农公交”、直达公交、观光公交等特色线路。“七五”普法全面启动。第十届村(居)民委员会换届选举圆满完成。深入开展安全生产大检查大整治，生产安全形势总体稳定。食品药品安全监管综合业务平台投入运行，6273家企业、983家学校食堂纳入在线监管。社会治安防控体系不断完善，信访法治化建设稳步推进，社会保持和谐稳定。

　　八是不断加强自身建设。我们围绕学懂、弄通、做实，深入学习宣传贯彻党的十九大精神。积极推进依法行政，深化“放管服”改革，启动相对集中行政许可权改革试点，全面实行“一窗受理”，审批办理提速91%。持之以恒正风肃纪，持续开展“嘉廉话”阳光问廉，重拳整治“微腐败”，“护根行动”经验在全省推广。保持反腐高压态势，处置问题线索2937件，立案968件，党纪政纪处分885人。自觉接受市人大及其常委会的法律监督、工作监督和市政协的民主监督，办结市人大代表建议、市政协委员提案641件，满意和基本满意率达100%。

　　各位代表：回顾过去一年的政府工作，成绩来之不易。这是省委、省政府和市委坚强领导的结果，是市人大、市政协和社会各界监督支持的结果，是全市广大干部群众共同奋斗的结果。在此，我代表市人民政府，向全市各族人民，向驻乐部队指战员、武警官兵、政法干警，向各民主党派、工商联、无党派人士、各人民团体和各界朋友表示衷心的感谢，并致以崇高的敬意!

　　在看到成绩的同时，我们也清醒地认识到，我市经济社会发展仍面临一些困难和问题，主要表现为：经济发展质量还不高，推进产业转型升级还有较大差距;新的财源税源增长点较为缺乏，财政收支矛盾突出;民生保障等方面还有短板和欠账，社会事业发展相对滞后等等。对此，我们高度重视，正在采取积极措施，认真加以解决。

　　2018年工作安排

　　2018年是贯彻党的十九大精神的开局之年，是改革开放40周年，是决胜全面建成小康社会、实施“十三五”规划承上启下的关键一年。市政府工作的总体要求是：全面深入贯彻落实党的十九大精神，坚定以习近平新时代中国特色社会主义思想为指导，深入实施省委治蜀兴川总体方略，牢牢把握稳中求进工作总基调，以供给侧结构性改革为主线，围绕“一个取向、四个定位、五个转变、六项重点工程”的总体思路，以“产业发展年”为主题，坚持质量第一、效益优先，着力强化项目支撑，加快产业升级，提高发展质量，保障改善民生，推动质量变革、效率变革、动力变革，打好防范化解重大风险、精准脱贫、污染防治攻坚战，奋力建设美丽繁荣和谐乐山。主要目标是：地区生产总值增长8%左右，全社会固定资产投资增长11.5%，规模以上工业增加值增长9.2%，社会消费品零售总额增长11.5%，地方一般公共预算收入增长8%，城镇居民人均可支配收入增长8%，农村居民人均可支配收入增长9%，常住人口城镇化率提高1.4个百分点，全面完成省下达的各项目标任务。我们将重点抓好以下工作：

　　一、突出项目投资拉动

　　立足打基础、利长远、惠民生，充分发挥投资对优化供给结构的关键性作用，统筹推进283个省市重大项目及35个挂图作战项目建设，夯实产业发展基础，助推实现高质量发展。

　　扩大产业投资。紧扣“产业发展年”主题，重点抓好总部经济建设、“三基地”建设、景区扩容提质等13个挂图作战产业化项目，完成产业化项目投资200亿元以上。突出工业转型发展和服务业倍增行动，着力引进落地一批技术领先、延链补链、绿色环保、税收贡献大的产业化项目，确保工业投资增长10%以上，服务业投资占比提高到68%以上，不断培育新的经济增长点。加快产业化项目建设进度，力争和邦10万吨蛋氨酸一期、峨眉钰泉30万吨矿泉水一期、凤生12万吨生活用纸技改等一批产业化项目竣工投产，尽快形成新的增量。

　　强化基础建设。打好“三年交通大会战”收官战，完成交通投资260亿元以上。开工乐山机场、岷江航电老木孔枢纽、龙溪口至宜宾航道整治等项目主体工程，加快岷江航电犍为和龙溪口枢纽、成昆铁路峨米段、成贵铁路、连乐铁路、成乐高速扩容、仁沐新高速井沐马段、峨汉高速、乐西高速等项目建设。统筹抓好省道308改线和215线过境公路、乐夹大道、眉乐快速干道等地方公路建设，建成乐峨大道、五犍沐快速公路沐川段。围绕山水园林宜居城定位，加快新型城镇化建设，切实提升城市品质，完成城建投资280亿元以上。重点加快苏稽新区、冠英新区路网建设，推动苏稽文体中心、乐山会展中心等项目落地，抓好高速出口乐山城区连接线、致江路桥、青衣江大道南段、通棉路等一批城市道路桥梁建设。统筹推进能源、水利、环保等基础设施建设，抓好沙坪二级水电站、青衣江河道综合治理、城市生活垃圾环保发电厂等项目，竣工市第四自来水厂。

　　抓好民生项目。坚持以群众需求为导向，持续加大民生投入，深入实施民生工程，办好民生实事。积极腾地改善办学办医条件，抓好乐山一中扩建、乐山二中迁建、市实验中学扩建等项目，确保乐山八中新校园建成投用，推进市人民医院新区医院、市妇幼保健院整体迁建、市疾控中心实验大楼、市中心血站业务大楼等项目建设。加强城市精细化管理，新改建主城区农贸市场12个，启动长江市场、牛咡桥市场搬迁工作，打通城区断节路5条，新增公共停车泊位8000个，全面完成主城区背街小巷整治。优化住房保障体系，推进保障性安居工程建设，实施棚户区改造8460户，基本建成9638套，竣工9998套。开展城区、景区、农村“厕所革命”，新改扩建公厕350座。保障便捷出行，创建“公交都市”，新投放和更换新能源公交车260台，争取开行乐山至周边城市旅游专列，推进成乐动车组公交化运营。

　　二、加快服务业发展倍增

　　着力构建以旅游业为主导的现代服务业体系，推动服务业发展提速、比重提高、效益提升，力争服务业增加值增长9.5%，占地区生产总值比重提高到40%以上。

　　突出旅游主导。做好旅游“三篇文章”，力争全年旅游综合收入、接待游客人次分别增长15%和10%。推进扩容提质，抓好乐山大佛南游客中心、乌尤坝禅修小镇、“只有峨眉山”实景演出、峨眉山张沟片区综合开发等项目建设，促进“两核一带”联动发展。推进景城一体，抓好“夜游三江”二期、凤洲坝旅游开发等项目，启动嘉定坊、花湖湾、就日峰改造提升工程，促进游江游山游城有机结合。推进全域旅游，支持千佛岩·东风堰、黑竹沟、大风顶、大瓦山、大峡谷、美女峰、竹海等景区建设，积极培育茶旅、康养、休闲等旅游新业态，鼓励嘉阳·桫椤湖、农夫山泉等景区上档晋级。大力发展“乐山造”旅游商品，整体提升研产销产业链，加快构建四川旅游商品集散地。

优化服务业态。培育特色会展经济，加快建设区域性会展中心城市，办好第五届旅博会、第三届茶博会。大力发展电子商务，深入实施“全企入网、全民触网、电商示范”工程，新创2个省级电子商务进农村示范县，力争电商交易规模突破350亿元。推进四川金融次中心建设，筹建乐山农商行、市商业银行科技支行，支持市商业银行增资扩股。打造物流信息平台，抓好专业园区、快递业、第三方物流等发展，促进物流业降本增效。优化商业网点布局，规划打造各类专业市场和商业综合体，改造提升餐饮、住宿、商贸等传统商业业态和品质。

　　强化平台支撑。以国家服务业综合改革试点为统揽，认真落实促进服务业倍增发展政策措施，创新行业管理、统计调查、财政金融等体制机制，充分发挥综合改革的牵引作用。做强企业主体，深入实施服务业“三百工程”，积极扶持个转企、小升规和主辅分离，新增规模以上服务业企业30家、限额以上单位及个体户60家。依托全国质量强市示范城市、国家信息消费试点市、全省放心舒心消费城市等创建平台，统筹推进质量对标、服务创新和诚信消费，不断优化消费环境，持续释放消费新潜力。

　　三、推动工业发展转型升级

　　围绕新兴产业培育和传统产业改造，发力推动存量变革、增量崛起、质量提升，以工业转型支撑现代化经济体系构建。

　　打造总部基地。围绕“两年见成效”目标，打好“一总部三基地”建设攻坚战，力争完成主营业务收入600亿元，工业集中度提高1个百分点。发挥高新区龙头引领作用，大力实施本土计划、归巢计划、巴蜀计划、比邻计划，加快总部经济区基础设施及配套项目建设，引进各类总部项目30个以上，延展“总部+基地”功能链条。支持五通桥基地加快发展光电信息、精细化工产业，抓好和邦、福华等企业精细盐磷化工项目，力争主营业务收入突破180亿元。支持犍为基地尽快提升基础承载能力，加快先进装备、新型材料等一批临港产业项目入园，力争主营业务收入突破100亿元。支持夹江基地以创建国家军民融合创新示范区为抓手，培育发展民用核技术、医用同位素、军民两用新材料产业，力争主营业务收入突破160亿元。

　　培育优势产业。优先发展光电信息、军民融合、新材料、特色轻工等新兴产业，确保新兴产业主营业务收入占规模以上工业比重提高1个百分点以上。依托多晶硅产能规模优势和产业配套基础，加快永祥“5+2+2”项目等建设，推动光伏全产业链发展，打造“中国绿色硅谷”。推动军转民、民参军，促进碳纤维航空复合材料、同位素溶液堆、石墨烯等一批项目落地见效，实现军民深度融合发展。做大光电信息体量，抓好希尔电子芯片规模化生产线、菲尼克斯半导体扩建等项目。大力发展食品饮料、医药健康等特色轻工产业，加快保乐力加威士忌、大冢制药西南地区总部等项目建设。抢抓数字经济发展新机遇，培育大数据、人工智能等新产业，推进未来科技产业园、中国网库电商谷等项目建设，提升新兴产业发展层次。持续改造提升传统产业，推进冶金建材向高端制品、盐磷化工向精细应用、农产品加工向高附加值方向转型发展，让老产业育出新产品，焕发新活力。

　　振兴实体经济。把经济发展的着力点放在实体经济上，构建亲清新型政商关系，大力发展民营经济，激发和保护企业家精神，夯实稳增长根基。坚持“一企一策”帮扶，引导困难企业加快技术创新、产品创新、品牌创新和组织创新，提升“乐山造”工业品市场竞争力，确保产销率保持在95%以上。实施中小微企业成长工程，梯度培育大企业大集团，新增规模以上工业企业60家，新培育主营业务收入20亿元以上企业2家、10亿元以上企业5家。打好实体经济降成本“组合拳”，积极开展基金合作、直接融资、风险分担、应急转贷等工作，力争享受直购电优惠政策企业超过200家，进一步降低企业综合成本。

　　四、着力实施乡村振兴战略

　　按照产业兴旺、生态宜居、乡风文明、治理有效、生活富裕的总要求，深化农业供给侧结构性改革，推动农业增效、促进农民增收、助力农村发展，加快农业农村现代化进程。

　　提高供给质量。突出规模化、标准化、品牌化、信息化方向，新培育各类新型农业经营主体100个，确保产业化带动面提高1个百分点。抓好建基地、创品牌、搞加工等重点任务，加快“一区六带”建设，促进质量兴农、绿色兴农。推进峨眉山茶区域品牌建设，打造高标准茶园5万亩，争创国家出口茶叶质量安全示范区。突出“高山蔬菜”“直供蔬菜”等重点，改造提升优质蔬菜面积5万亩。积极发展中药材产业，新建道地和特色优势中药材基地7000亩。支持发展优质杂交柑橘等水果产业。大力发展原产地初加工，促进传统养殖业转型升级，提高附加值。完善农产品标准、监测、认证和质量安全监管体系，新增“三品一标”农产品30个。推进国家农业科技园区建设，做强做大绿创科技园区公司，培育园区知名品牌5个以上。持续抓好粮食安全工作，深入推进“优质粮食工程”建设，新建高标准农田14.2万亩，稳定粮食总产量。

　　促进农民增收。深化农业农村改革，健全城乡融合发展体制机制，清除阻碍要素下乡各种障碍。落实农村土地集体所有权、农户承包权、土地经营权“三权分置”制度，加快建设农村产权流转交易平台，力争新增土地流转面积3万亩，适度规模经营率提高到23%。依托“县乡村特色品牌创建工程”，因地制宜发展农村一二三产业融合模式，引导有条件的农民发展乡村旅游、民宿旅游、休闲农业等特色产业，实现增收致富。加快发展农村电商，完善配套服务和运作模式，力争乡(村)电商服务站突破1000个，提升农产品商品率，让农民通过线上销售获取更多收益。强化政策配套，全面落实各项强农惠农富农政策。开展新型职业农民培育试点，培养一批爱农业、懂技术、善经营的新型职业农民，提高就业增收能力。

　　建设美丽乡村。统筹抓好城乡规划编制，保护好传统村落，建成幸福美丽新村150个。加快农村基础设施建设，新改建农村公路1600公里，全面推进渡改公路桥建设，持续改造升级农村电网，推动天然气向有条件的农村地区覆盖，促进城市公共服务向农村有效延伸。实施村容村貌整治，推进农村垃圾、生活污水和面源污染治理，保护山水田林湖生态。健全自治、法治、德治相结合的乡村治理体系，加强农村社区建设，培育乡风文明，办好农民夜校，给予农村留守儿童、妇女和老人更多关心和照顾。

　　五、打好脱贫攻坚硬战

　　聚焦“两不愁、三保障”和“四个好”目标，念兹在兹、唯此为大，百倍用心、千倍用力，下足“绣花”功夫，在去年再战再胜基础上推进脱贫攻坚后三年决战决胜。

　　聚力精准脱贫。严格落实“六个精准”要求，加大投入、对标补短，精准实施好年度扶贫专项，确保沐川县脱贫摘帽通过国家验收，87个省定贫困村和35个市列贫困村退出，3万贫困人口脱贫。继续改善住房及基础设施条件，因人因户配套落实产业就业、社会保障等措施，新改建农村危旧房2610户，完成易地扶贫搬迁12612人。深化“百企帮百村”，带动发展致富产业，扩大群众惠及面。发挥好四项扶贫基金作用，加大金融扶贫力度，新发放扶贫小额信贷1亿元以上。继续落实“一村一幼”“十免四补助”等教育健康扶贫政策，加快扫除村卫生室达标盲区。

攻坚深度贫困。坚持问题导向，集中优势兵力，坚决打赢小凉山地区脱贫攻坚硬仗中的硬仗，大幅降低彝区贫困发生率。统筹倾斜项目、资金、人才等帮扶资源，完善扶贫专项支持政策，确保金口河高标准高质量脱贫摘帽。把峨边、马边作为坚中之坚，突出重点任务和薄弱环节，着力交通扶贫，助力产业扶贫，推进旅游扶贫，补齐彝区发展短板。用好贫困地区城乡建设用地增减挂钩、企业上市绿色通道、“飞地经济”等政策，支持小凉山彝区与工业基地共建飞地园区。

　　凝聚帮扶合力。完善脱贫攻坚考核体系，加强扶贫领域执纪问责，精准落实脱贫不脱政策、不脱帮扶、不脱项目，构建稳定脱贫长效机制。拓展浙川东西扶贫协作，扩大经贸、人才、技术等帮扶成果，确保一批基础设施、产业培育、生态保护等帮扶项目落地落实。深化“1+3”对口援彝，帮助美姑县建设2个扶贫新村，加快推动循环农业生态养殖等项目建设，同步做好市内三对区县对口帮扶。建强蹲点帮扶干部、驻村工作队、村第一书记队伍，确保领导联系全覆盖、包村包户全覆盖。深入推进励志扶贫，持续开展感恩奋进主题宣讲、贫困家庭奋进计划、“四好村”创建等活动，营造比学赶帮超氛围，激发群众内生动力。

　　六、持续深化改革开放

　　以纪念改革开放40周年为契机，坚定改革定力，坚持开放态度，推动新旧动能接续转换，为转型发展提供源源不断动力活力。

　　发力重点改革。坚持一手抓方案制定，一手抓任务落实，出台创新改革方案10个以上，做实改革主框架。深化供给侧结构性改革，扎实推进“三去一降一补”，大力破除无效供给，积极处置“僵尸企业”，推动化解过剩产能，降低企业杠杆率。深化国家级旅游业改革创新先行区和全省旅游综合改革试点工作，全面完成各项规定任务、商定任务和自选任务。深化国资国企改革，持续完善“1+N+n”国有资本管理体系，新组建农投等国企集团，积极推进国企集团信用评级，力争完成融资120亿元，促进国有资产保值增值。推进政府与社会资本合作，激发社会投资动力，支持有实力的企业集团参与市政基础设施建设。深化商事制度改革，加快企业信用体系建设，激发各类市场主体活力。推进税收制度改革，建立现代财政制度，壮大税源经济，加强预算执行管理和地方政府债务管理。

　　推进全面开放。坚持引进来和走出去并重，积极参加中外知名企业四川行、第十七届西博会、千企丝路行、万企出国门等投资促进及经贸合作活动，精准开展延链招商、补缺招商、以商招商，提高项目签约落地率，确保到位市外内资600亿元以上。拓展与“一带一路”沿线国家和地区的多元合作，深化与京津冀、长三角、珠三角、成渝等经济区的务实合作，积极承接家居家具、智能终端等产业转移。加强与国内外知名组织、城市、景区的交流互动，力争新增国际友好城市2个。推进保税物流中心(B型)、国检试验区等建设，营造市场化、国际化、法治化营商环境，力争进出口总额突破10亿美元。

　　促进创新创业。深入实施创新驱动发展战略，加强创新体系建设，打造创新型城市，确保科技进步综合指数水平提高1个百分点。大力发展高新技术产业，推进企业创新主体建设，促进科技成果转化，新建特种钢等一批新型产业技术研究院，力争新培育高新技术企业15家、科技企业孵化器2家。推进省级科技金融结合综合试点建设，引导科技资源与金融资源高效聚集，打造“绿创动能”科技金融结合特色模式。倡导创新文化，加大知识产权创造、保护和应用力度，新增专利实施项目300个，争创国家知识产权试点城市。统筹实施企业“双创”示范、乡村“双创”兴业、“乐创汇”回引等工程，优化就业结构、提高就业质量，确保城镇新增就业3.8万人。推进“嘉州英才工程”，全面加强创新人才和创新团队建设。

　　七、深入推进生态文明建设

　　坚定不移把生态文明建设和环境保护作为“生命工程”来抓，加快构建适应绿色发展的空间体系、产业体系、城乡体系和制度体系，让嘉州大地天更蓝、山更绿、水更清、环境更优美。

　　强化生态保护。落实主体功能区规划，加强世界遗产地、自然保护区、风景名胜区等重点区域生态管控，划定并严守生态保护、永久基本农田、城镇开发边界三条控制线。深入实施绿秀嘉州行动，统筹抓好岷江大渡河森林质量精准提升、多彩通道、退耕还林还草等工程建设，完成营造林55万亩，确保森林覆盖率提高到57.52%。继续实施“三百水利工程”，全力打造“一湖五湿地”，加快建设竹公溪湿地、大渡河湿地，开工建设三江湿地和峨眉河湿地，推进“引青济峨”工程。依法加强绿心保护，抓好绿心生态整治项目。加强生态文明体制机制建设，建立国有自然资源资产管理和自然生态监管机制，完善环境评价、生态补偿、排污许可、总量控制等制度，推进饮用水水源保护、景区管理等地方立法，全面完成县级饮用水水源地保护规范化建设。

　　防治环境污染。深入实施污染防治“四大战役”，扎实抓好中央和省环保督察反馈问题整改，强力推进突出环境问题整治，不断改善生态环境质量。打好蓝天保卫战，落实能源结构调整、中心城区禁燃烟花爆竹、重型柴油车限行管控、道路扬尘治理、大气污染联防联控等措施，推行水泥、钢铁、化工、铁合金、制浆造纸、焦化等行业特别排放限值改造，完成大中型燃煤锅炉淘汰或改造42台，促进空气质量持续改善。打好碧水保卫战，深入推进河长制，全面消除主城区及景区周边黑臭水体，打造茫溪河治理示范工程。实施城镇污水治理攻坚，推进乡镇污水处理设施和主城区截污管网全覆盖，消除市县城区生活污水直排口。打好净土保卫战，完成全市垃圾处理场整治，抓好农用地土壤污染治理与修复试点、医疗废物处置、餐厨垃圾处理等项目建设。开展第二次全国污染源普查和土壤污染状况详查。

　　发展低碳经济。强化绿色产业支撑，加快盐磷化工循环、节能环保装备、清洁能源、清洁生产等产业发展。积极实施燃气替代、电能替代、清洁生产万家企业节能低碳等行动，降低能耗、物耗和污染物排放水平，确保完成省下单位地区生产总值能耗下降目标。大力发展清洁农业，积极推广生态循环种养模式。多管齐下做好绿色“加法”，培育生态文化，倡导绿色消费，引领全民为保护环境尽一份心、出一份力、担一份责。

　　八、切实保障和改善民生

　　坚持把最广大人民根本利益作为最高标准，在发展中补齐民生短板，增强群众获得感幸福感安全感，不断满足全市人民日益增长的美好生活需要。

　　促进文化繁荣。坚定文化自信，大力践行社会主义核心价值观，开展全国文明城市创建。完善公共文化服务体系，广泛开展群众文化活动，抓好一批村文化室、农家书屋、本地电视节目全覆盖等文化惠民工程，完成市文化馆、市图书馆改造，确保通过国家公共文化服务体系示范区验收。发展文化创意、影视演艺等文化产业，创作更多本土文艺精品力作，打造嘉州长卷戏剧文创特色街区，办好第三届佛光花海音乐节。加强文物保护利用，抓好老城区历史文化遗址保护挖掘，推进战时故宫文物南迁遗址公园建设。促进文化旅游深度融合，打造佛禅文化、武术文化、沫若文化、小凉山彝族文化等特色文化品牌，筹建峨眉武院。

优先发展教育。深化教育教学改革，稳妥接轨高考制度改革，加强教师队伍和师德师风建设，提高各级各类学校教育质量。实施第三期学前教育行动计划，新改扩建幼儿园39所，增加普惠幼儿学位4000个。协调发展普通教育，加快消除中小学“大班额”现象，分别完成义务教育均衡发展3个县国家验收认定和3个区县省级督导评估。积极发展职业教育，深化产教融合和校企合作，推进中职学校达标建设。抓好大学城基础设施建设，实施乐山师院新校区等高校发展项目，积极引进知名学校和社会力量兴办基础教育。

　　打造健康乐山。深化医药卫生体制综合改革，健全全民医保制度，推进分级诊疗、家庭医生签约服务等工作，实行医疗医保医药联动，新建医疗联合体20对。鼓励社会力量办医，增加医疗卫生资源供给，补好产科、儿科、全科医生等短板。积极发展大健康产业，完善社会化养老服务体系，加强食品药品监管，鼓励中医治未病、养老养生中心等项目落地，建设全省医养结合试点市。加强人口和计生管理服务。深化爱国卫生运动，争创国家卫生城市。广泛开展全民健身活动，新增城乡公共健身设施200个，举办2018乐山国际马拉松赛，申办省第十四届运动会。

　　促进和谐稳定。深入推进依法治市，认真实施“七五”普法规划，建立完善市县乡公共法律服务平台。加强社会治安综合防控，严厉打击暴恐活动，持续推进网络安全、禁毒防艾等专项整治，完成主城区公安高清视频系统和农村“雪亮工程”建设，建成市县乡村四级综治中心。完善安全生产责任制，认真做好群众来信来访工作，积极防范金融风险，提高应急响应、救援救灾等能力，坚决守住安全稳定底线。加强国防和后备力量建设，巩固全国双拥模范城成果。加强社会保障体系建设，抓好城乡低保等社会救助工作，动态调整保障标准。积极发展妇女儿童、残疾人、老龄、慈善、关心下一代等事业。做好第四次全国经济普查，统筹抓好民族宗教、对台外侨、防震减灾、地方志、保密、档案、气象、人防等工作。

　　九、建设人民满意的服务型政府

　　中国特色社会主义进入新时代，对政府工作提出了新的更高要求。我们将进一步转变政府职能，深化简政放权，不断增强政府公信力和执行力，更好地为人民群众服务。

　　强化政治建设。坚持把学习宣传贯彻党的十九大精神作为当前和今后一个时期首要政治任务，引导广大公职人员认真学习领会习近平新时代中国特色社会主义思想深刻内涵，牢固树立“四个意识”，切实增强“四个自信”，自觉在政治立场、政治方向、政治原则、政治道路上同以习近平同志为核心的党中央保持高度一致。大力弘扬“红船精神”，开展好“不忘初心、牢记使命”主题教育，推进政府系统“两学一做”学习教育常态化制度化。认真贯彻民主集中制原则，自觉接受市人大、市政协和社会各界监督，健全落实政府重大决策出台前向人大报告工作制度。自觉遵守党章和各项规章制度，严格执行新形势下党内政治生活若干准则，营造风清气正的良好政治生态。

　　提升政府效能。纵深推进依法行政，深化行政执法体制改革，严格规范公正文明执法。持续推进机构改革，探索市县职能相近的政府机关合并设立或合署办公，推进事业单位政事分开、事企分开、管办分离。深入推进“互联网+政务服务”，完善一体化政务服务平台建设，建成市级数据共享交换平台。打造城市管理服务中心，启用“12345”一号通综合服务平台。深化相对集中行政许可权改革试点。始终把抓落实作为政府工作的首要任务，坚持说实话、谋实事、出实招、求实效，雷厉风行、攻坚克难、久久为功，以钉钉子精神做实做细做好各项工作。

　　坚持反腐倡廉。深入贯彻中央八项规定实施细则精神，认真落实省市十项规定实施细则要求，坚持纠正“四风”不止步，坚决防止不良风气反弹回潮。认真落实党风廉政建设“两个责任”，配合做好监察体制改革，实现对所有行使公权力的公职人员监察全覆盖。进一步深化“护根行动”，加大整治群众身边腐败问题力度，凡是群众反映强烈的问题都要严肃认真对待，凡是损害群众利益的行为都要坚决纠正。坚持无禁区、全覆盖、零容忍，坚持重遏制、强高压、长震慑，保持惩治腐败定力，保证干部清正、政府清廉、政治清明。

　　各位代表：站在新时代，要有新气象，更需新作为。让我们更加紧密地团结在以习近平同志为核心的党中央周围，高举习近平新时代中国特色社会主义思想伟大旗帜，在省委、省政府和市委的坚强领导下，不忘初心、牢记使命，锐意进取、埋头苦干，为加快建设美丽繁荣和谐乐山、决胜全面建成小康社会、实现人民对美好生活的向往而不懈奋斗!
